# Supplementary figures and images for: Sequential mutations in exponentially growing populations
Source: PLoS Comput Biol. 2023 Jul 10;19(7):e1011289. doi: 10.1371/journal.pcbi.1011289 (PMC10359018; doi:10.1371/journal.pcbi.1011289)

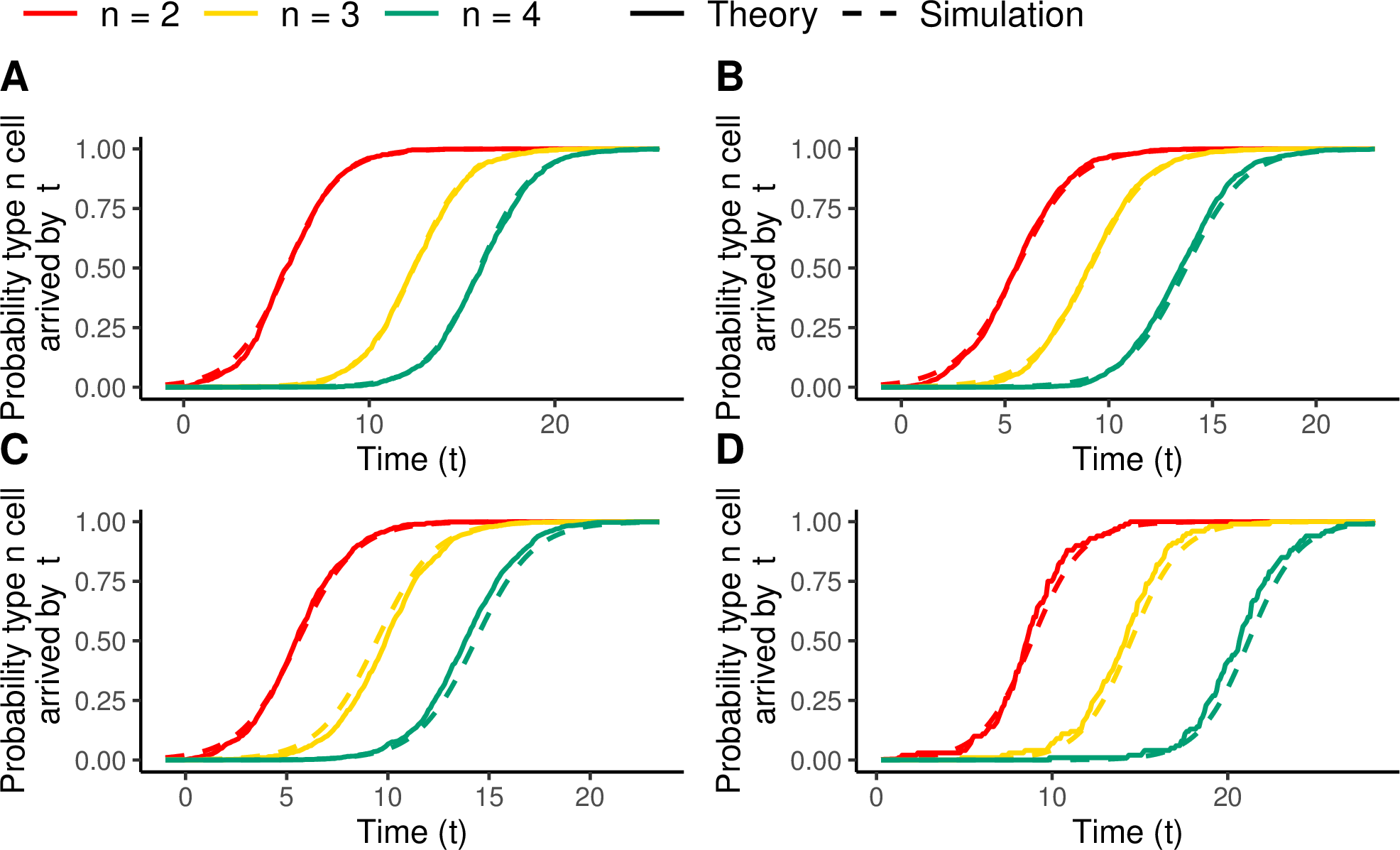

Supplement: S1 Fig — Empirical cumulative distribution of the arrival times of types 1–3 obtained from simulations of the exact model versus the cumulative distribution function corresponding to the logistic distribution of Eq 4. Birth/death parameters: A (net growth rate decreases then increases), α1 = α2 = 1, α3 = 1.4, β1 = β3 = 0.3, β2 = 1.5; B, D (net growth rate increases then decreases); α1 = α3 = 1, α2 = 1.4, β1 = β2 = 0.3, β3 = 1.5; C (neutral), α1 = α2 = α3 = 1, β1 = β2 = β3 = 0.3. Mutation rates: A, B, C, ν1 = ν2 = ν3 = 0.01; D, ν1 = ν2 = ν3 = 0.001. Number of simulations: A, B, C; 1000 simulations; D, 100 simulations. (TIF) [file pcbi.1011289.s001.tif]
