# Supplementary material for: Sequential mutations in exponentially growing populations
Source: PLoS Comput Biol. 2023 Jul 10;19(7):e1011289. doi: 10.1371/journal.pcbi.1011289 (PMC10359018; doi:10.1371/journal.pcbi.1011289)
Supplement: S1 Text — (PDF) [file pcbi.1011289.s002.pdf]

Supplementary material: Sequential mutations in exponentially growing populations

Michael D. Nicholson[1*], David Cheek[2], Tibor Antal[3]

[1]Edinburgh Cancer Research, Institute of Genetics and Cancer, University of Edinburgh
[2]Center for Systems Biology, Department of Radiology, Massachusetts General Hospital Research Institute and Harvard Medical School
[3]School of Mathematics and Maxwell Institute for Mathematical Sciences, University of Edinburgh

June 28, 2023

1 Statistical methods for n-mutation fluctuation assay

For the fluctuation assay simulations presented in Fig. 4, we performed simulations of a 3-type birth-death-mutation process with $\alpha_i = 1$, $\beta_i = 0$, and $\log_{10}(\nu_i)$ either $\{-3, -2.5, -2, -1.5\}$ for each i. For each mutation rate 100 simulations were performed, simulations were stopped at $t = 10$ and the number of type 3 cells were recorded (mutant counts), which were assumed to be the cells resistant to a given therapy. The simulated data was then used to infer the underlying mutation rate using either the p_0 method or maximum likelihood on the mutant counts as follows.

For the p_0 method, observe that the number of simulations yielding no type 3 cells is binomially distributed with 100 replicates and success probability $\mathbb{P}(\tau_3 > 10)$ (where we used the simulation stopping time of $t = 10$). Eq. 4 gives an approximation for $\mathbb{P}(\tau_3 > 10)$ and so using this approximation, for given parameter values the likelihood of the data (number of simulations with no type 3 cells) may be numerically evaluated. For maximum likelihood on the mutant counts, the distribution for the number of type 3 cells is approximated for large times by Eq. 1. If $Z_3^{(k)}(10)$ is the number of type 3 cells in the kth simulations, then

$$\frac{Z_3^{(k)}(10)}{10^2 e^{10}}$$

*mdnicholson5@gmail.com

is Mittag-Leffler distributed with tail parameter 1 and scale parameter ω_3 which is numerically obtainable via the recursion of Eq. 2. As the simulation parameters were chosen as $\alpha_i = 1$, $\beta_i = 0$ for $i = 1, 2, 3$, then for type 3 the running-max fitness and number of times it has been attained are $\delta_3 = 1$ and $r_3 = 2$. Thus, the random amplitude V_3 follows a Mittag-Leffler distribution with tail parameter 1, and so is an exponential distribution with mean as the scale parameter ω_3. Hence for given parameter values, with f_{V_3} as the density of the relevant Mittag-Leffler distribution, the likelihood of the mutant counts over the 100 simulations is

$$\prod_{k=1}^{100} f_{V_3}\left(\frac{Z_3^{(k)}(10)}{10^2 e^{10}}\right).$$

For both approaches, numerical likelihood values were obtained over a grid of $\log_{10}(\nu_i) \in [-4.5, 0]$ with grid steps of 0.01. The mutation rate that achieved the highest likelihood values is reported as the maximum likelihood estimate (mle). 95% confidence intervals were obtained by finding the mutation rate such that the normalised log-likelihood value (log-likelihood of data at given mutation rate - log-likeliood of data at the mle) dipped below -1.92, in accordance with the likelihood ratio test (see page 47 of Ref. [1]).

References

[1] Pawitan Y. In all likelihood : statistical modelling and inference using likelihood / Yudi Pawitan. Oxford: Clarendon Press; 2001.
