# Supplementary material for: Sequential mutations in exponentially growing populations
Source: PLoS Comput Biol. 2023 Jul 10;19(7):e1011289. doi: 10.1371/journal.pcbi.1011289 (PMC10359018; doi:10.1371/journal.pcbi.1011289)
Supplement: Attachment — Submitted filename: Review.pdf [file pcbi.1011289.s003.pdf]

Review for ”Mutation accumulation in exponentially growing populations”

Nicholson et al. studied the probability distributions of the arrival time and abundance of mutants in an exponential growing population. They reported the shapes of these distributions are independent from the selection strength and the number of the mutations under small mutation rates.

My main question is about the linear evolutionary path assumption. The definition of net growth rate seems to be specific for type n cells with n particular mutations. This differs from standard random mutation accumulation processes, where cells with n mutations can have different combinations of mutation set. The linear evolutionary path seems to be a strong assumption related to a very specific regime of driver mutations, where a new mutation only arises in cells containing all previous mutations. Consequently, the rate of new mutation arising in the whole population is not related to the total population size or total cell numbers under reproduction, but only to the sub population size of the last mutant. It might appears in biological data that driver mutations are detected one after another over time, which is an outcome under the balance of selection and fixation. But I think this is different from the assumption of the sequential acquisition of driver mutations in this model. It would be clear if the authors can cite some experimental papers beside of pervious theoretical models to specify under which context this assumption is relevant. It makes it hard to understand the generality of the results without understanding the motivation of this assumption.

Alternatively If possible, it would be good to know under which condition a standard natural mutation accumulation process would lead to the linear evolutionary path patterns as in this paper.

Some results are obtained under certain special conditions compared to the general set up of this model. Can you group those results with more clear subtitles? For example, I was not sure whether eq 3) still belongs to the condition that ” type 1 has the maximal growth rate of all types up to type n, that is $\delta_n = \lambda_1$”.

Relatedly, it is better to add some intuitive explanation of some conditions, e.g. ”If we assume each running-max fitness is attained only by one type ($r_i = 1$ for each i)”. If we think about a curve representing the net growth rates of all types up to type n with the x-axis representing type 1, 2, ... n, what kind of shapes such a curve should be to fulfil this condition? What does it mean biologically?

Fig 2 shows some stochastic simulations under certain λ_1/δ_2 values constructed by given α_i, β_i, ν_i. Does the concrete choice of α_i, β_i, ν_i have any impact on outcomes of the stochastic simulations?

Minor Comments:

1. There are many notations. I was rolling up and down to follow them. It might be helpful to have a table of all notations.

2.Page 8 Figure 1 caption: ”Grey line indicates where the population sizes” . I think you did not mean the total population size here. This can be more specified.

3.. The definition of V_n does not follow Eq. 1), where it is first time introduced.

4."Similarly to the population sizes, the exact distribution of the arrival time is analytically intractable outside of the simplest settings." what are the simplest settings?
5. Can you add the details of the stochastic simulations in the method part?
