# Supplementary material for: Sequential mutations in exponentially growing populations
Source: PLoS Comput Biol. 2023 Jul 10;19(7):e1011289. doi: 10.1371/journal.pcbi.1011289 (PMC10359018; doi:10.1371/journal.pcbi.1011289)
Supplement: Attachment — Submitted filename: PLOS_CB_Referee_Report_final.pdf [file pcbi.1011289.s004.pdf]

Referee report, *PLOS Computational Biology*, manuscript PCOMPBIOL-D-22-01344
Title: Mutation accumulation in exponentially growing populations
Authors: M.D. Nicholson, D. Cheek, T. Antal

In this manuscript, the authors study a multitype branching process model of mutation accumulation in an exponentially growing cell population. In the model, type-n cells can divide, die or mutate to type-$(n+1)$ at rates α_n, β_n and ν_n, respectively. The authors present mathematical results on (i) the size of the type-n population $Z_n(t)$ at time t, and (ii) the arrival time τ_n of the first type-n cell. For the case where mutations do not lead to fitness increases, $\lambda_i \leq \lambda_1$ for all $i = 1, \ldots, n$, a distribution for $Z_n(t)$ is given for large t and general $\nu_1, \ldots, \nu_{n-1}$, and a distribution for τ_n is given in the limit of small mutation rates (the ordered limit $\lim_{\nu_1 \to} \cdots \lim_{\nu_{n-1} \to 0}$). For the alternative case $\lambda_i > \lambda_1$ for some i, the authors turn to an approximate version of the model. In this model, the emergence of type-$(i+1)$ cells is studied under the assumption that the type-i population grows according to its long-run trajectory. For example, if $Z_1(t)e^{-\lambda_1 t} \to V_1$ as $t \to \infty$ for type-1 cells, type-2 cells are produced at rate $\nu_1 V_1 e^{\lambda_1 t}$ at any time $t \geq 0$. The authors compute the distributions of $Z_n(t)$ for large t and τ_n under the approximate model, in the limit of small mutation rates. An analogous approximate model has been previously applied by Durrett and Moseley to study the case $\lambda_1 < \lambda_2 < \cdots < \lambda_n$.

The main text consists of a summary and discussion of the mathematical results, followed by a brief discussion of their potential application to mutational dynamics in bacteria and cancer. The methods section, which is around twice as long as the main text, contains proofs of the mathematical results. While I think the manuscript is generally well written and the results are interesting, I have the following main concerns:

1. The goal of the paper is to study the distributions of the size of the type-n population and the time of first appearance of a type-n cell in a general setting. The results are discussed as if they hold for any birth and death rates, and Figures 3 and 4 show examples where $\alpha_i < \beta_i$ for some i. However, the proofs seem to assume that each cell type follows a supercritical branching process, with $\alpha_i > \beta_i$ for all i.
2. For the $\lambda_1 < \lambda_2 < \cdots < \lambda_n$ case, the main results of the paper have already been established by Durrett and Moseley. Insights emphasized in the present manuscript, such as the fact that the shape of the distribution of τ_n is the same for all n, and the fact that the time between new mutants appearing shortens with increasing n for the case $\lambda_1 < \lambda_2 < \cdots < \lambda_n$, have been noted before. The novelty of the results and insights should be addressed more clearly, and the formulas obtained should be compared directly with the formulas obtained by Durrett and Moseley.
3. The authors structure the paper in a way to make it more accessible to non-mathematical readers. However, there is still room for removing technical language, adding context and providing intuition. I make some suggestions below.
4. Irrespective of the last comment, I am not sure this manuscript is appropriate for PLOS CB. The mathematical results and their proofs are the meat of the

manuscript, whereas the application section lacks development. One potential way of addressing this is to flesh out more concretely how the results can be useful for parameter inference or analysis of data, and to preferably show that the ideas work on simulated or real data. A stronger case for the utility of the results seems especially important given that the mathematical contribution and related insights seem to largely extend or reemphasize prior work.

5. The manuscript should be read more carefully. I found a significant number of apparent typos, most of them in mathematical expressions.

I make more comments below, split into "major comments" (most of which elaborate on the above comments), "minor comments", and "typos".

Major comments.

1. Abstract: Referring to the distributions of the number and arrival time of type-n cells, it is said that: "Surprisingly, these distributions are universal in that their shape does not depend on the number or selective effect of the mutations considered." This doesn't seem to be true for the distribution of the number of type-n cells, which requires knowledge of the entire sequence $\lambda_1, \ldots, \lambda_n$.
2. P4, paragraph 1: I suggest expanding on the discussion of the result in (1). For example, point out explicitly that when $\lambda_n > \delta_{n-1}$, the growth is exponential with rate λ_n, while there is a geometric factor $t^{r_n - 1}$ when $\lambda_n \leq \delta_{n-1}$. In addition, provide intuition for why the geometric factor appears and why the exponent is $r_n - 1$.
3. P5, paragraph 1: I suggest expanding on the comment "For $\gamma < 1$ the density has a $x^{\gamma-1}$ singularity at the origin and a $x^{-\gamma-1}$ tail." Discuss the properties of the Mittag-Leffler distribution at a higher level, interpret what these properties mean for the distribution of $Z_n(t)$, and explain why the model dynamics produce a limiting distribution with these properties.
4. P7, paragraph 1: The comment on identifying whether a phenotype of interest requires 2 or 3 mutations based on fluctuations in τ_n presupposes that the distribution of τ_n is observable. Is that the case? Are there other observables that could be used to infer how many steps are required to produce a given phenotype, when combining the theoretical results with data?
5. P7, paragraphs 2-3: I suggest rewriting these paragraphs using more informal language and adding explanations. For example, I am not sure the following sentence is easily parsed by the general reader: "Then, solving for $t_{1/2}^{(2)}$ in $e^{\lambda_1 t_{1/2}^{(2)}} = 1/\nu_1$, produces the leading order as $\nu_1 \to 0$ (to be compared with Eq. (5))".
6. P7, paragraph 4: In the first application example, an experiment of multidrug resistance is suggested to infer the product $\prod_{i=1}^{n} \nu_i$ of the rates $\nu_1, \ldots, \nu_n$ for n sequential resistance mutations. I am not sure how realistic this example is. For example, in the hypothetical experiment, what is the time scale of waiting for n

sequential mutations? Do the cells need to be repassaged, meaning uninterrupted exponential growth cannot be assumed? Does the appearance of the $\log(\nu_n^{-1})$ factor in $t_{1/2}^{n+1}$ interrupt inference of $\prod_{i=1}^n \nu_i$? Even if $\prod_{i=1}^n \nu_i$ is theoretically identifiable by inverting expression (4), how confidently can it be inferred from real data?

7. P8-P9: In the application examples (ii)-(iv), arrival times of mutations are discussed depending on whether the mutations are advantageous, neutral or deleterious. The qualitative insights are for example that for $\lambda_1 < \cdots < \lambda_n$ and $\nu_1 = \cdots = \nu_n$, the time between the first type-n and the first type-$(n+1)$ cell decreases as n increases, and that the time between the arrival of a new mutant and the mutant growing to a detectable size is greater if the mutation is neutral or deleterious than if it is advantageous. These insights are not necessarily surprising, and in general the examples do not give concrete suggestions of how the theoretical results can be useful for example for statistical inference or analyzing data.
8. P11: In Lemma 4.2, which is used to prove Proposition 4.1, it is explicitly assumed that $\alpha > \beta$, which is not reflected in the statement of Proposition 4.1.
9. P17: In stating the limit of the birth-death process $Y(\cdot)$ and its Laplace transform, it is implicitly assumed that $\alpha_{n+1} > \beta_{n+1}$, but this assumption does not appear in the statement of Corollary 4.6 or discussions of this and related results.

Minor comments.

1. P3, paragraph 2: It could help to explain what the rates mean, for example in terms of the probability of something happening in a small time interval, since not every reader may be familiar with the continuous-time branching process framework.
2. P3, paragraph 5: I suggest replacing the semicolon with a period.
3. P3, paragraph 5: Immediately before expression (1), I suggest adding something like "and how many times it has been obtained up to type n" after "by the running max fitness".
4. P4, paragraph 2: I recommend showing the Laplace transform before introducing the distribution as Mittag-Leffler, since many readers may not know this distribution (including myself). Similarly, on page 5, I would show expression (4) before I call the distribution logistic.
5. P4, paragraph 2: My context-free interpretation of "fluctuations in cell number" is fluctuations over time, as opposed to variability at a fixed time across many tumors. I wonder if there is a better phrase which lends itself more to the second interpretation (referring for example to the "randomness", "stochasticity" or "variability" in cell number), or if it's worth it to explicitly make this distinction in the text.
6. P5, paragraph 3: Is it not relevant to also discuss the time to the first type-n which does not die out?
7. P7, paragraph 3: This paragraph is somewhat out of place. I recommend moving it so that it appears closer to the statement of the relevant result (1) and before you

start discussing the results for the time to the first type-n. I also suggest splitting the "Results summary" section into two subsections, one on the number of type-n cells at time t, and the other on the arrival time of type-n.

8. P7, paragraph 3: At the end of the paragraph, do you mean $\delta_n - \lambda_{n+1} \ll 1$ as opposed to $\delta_n - \lambda_{n-1} \ll 1$? It's not necessarily accurate to call the n-th mutation a deleterious mutation in this case, since we might have $\lambda_n < \lambda_{n+1} < \delta_n$.
9. P10, caption of Figure 4: The end of the caption is confusing, where β_n is set to $1 + ns_d$, whereas earlier in the caption β_1 is fixed to 1.
10. P11: In the statement of Proposition 4.1, the conditioning on nonextinction of type-1s should be explicitly stated.
11. P11: In the statement of Lemma 4.2, and in other similar cases, it would help to state that $Y_i(0) = 1$.
12. P11: As far as I can tell, there is no argument for the positivity of V in the proof of Lemma 4.2.
13. P12: Add "for $s \leq t$" before the martingale calculation.
14. P12: When giving the result for the second moment of a birth-death branching process, say where within Harris the result can be found.
15. P15: Proposition 4.4 would be more appropriately called a corollary.
16. P16: If you condition on V_n^*, is the resulting process not simply a Poisson process? Is there any reason to use the term "Cox process"?
17. P17: In the statement of Corollary 4.6, why not simply write $(r_n - 1)!$ instead of $\Gamma(r_n)$?
18. P17: Does the proof of Corollary 4.6 not use the same argument as in the proof of Theorem 3 in Durrett and Moseley? If so, it should be acknowledged.
19. P17: The derivation of the Laplace transform in (10) at the beginning of the proof is a little confusing. $Z_{n+1}(t)$ is a compound Poisson random variable, but it requires a little thought to see that it can be written as a sum of i.i.d. random variables. It would probably help to provide a more explicit calculation showing how one goes from the definition in (8) to the Laplace transform given in (10). A similar comment applies to the proof of Lemma 4.2, where it would also be helpful to show how $Z(t)$ can be written as a sum of i.i.d. random variables.
20. P18: For the integral representation of the Lerch transcendent to converge, I think you also need $\mathrm{Re}(s) > 0$ and $\mathrm{Re}(a) > 0$ to avoid problems at 0 and ∞.
21. P18: "Recall that it was heuristically argued that the random amplitudes of the approximate and original models coincide in the limit of small mutation rates". On page 16, it says that "We hypothesize but do not prove that ...", but I don't see a heuristic argument.
22. P19: It would help the reader a lot to number the definition of κ_n and refer to it when it is used.

23. P19: In the statements of Proposition 4.7, Lemma 4.10 and what follows, why not write α_1/λ_1 instead of $(1-\beta_1/\alpha_1)^{-1}$? (This is done in the main text when writing down the recursion for ω_n.)
24. P19: Is there no standard reference for Lemma 4.8?
25. P20: In the statement of Lemma 4.9, before showing the three cases, it would help the reader a lot to write the result more compactly as

$$h_n(f_n(\nu_n)^{1/\gamma_n}\theta) = \theta^{\gamma_n}\kappa_n.$$

    Having this form prominently displayed in the statement of the lemma helps when reading the proof of Proposition 4.7 on page 21.
26. P21: What are called terms at the top of the page are factors.
27. P22: I would suggest using a letter other than V_{n+1}, since V_{n+1} is used in a different context in the statement of the earlier Proposition 4.1.
28. P22: In the calculations for ω_{n+1} in the proof of Lemma 4.10, it would be more logical to reverse the two steps taken. The same comment applies to the calculations for ω_{n+1} on the following page.

Typos.

1. General: There are multiple places where punctuation marks are off (a period is missing, a comma appears where a period should be, etc.).
2. General: There are frequently double spaces following a period which does not end a sentence. For example, see the space between i.e. and δ_n on page 3, paragraph 3. This can be avoided by using tilde in the Latex code: "`i.e.~$\delta_n$`".
3. P3, paragraph 3: In the definition of r_n, there is a typo in $i = 1, \ldots, n$.
4. P4, caption of Figure 1: "with dashed line" should probably be "with dashed lines".
5. P5: In the top line, $n > 1$ should be $n \geq 1$.
6. P7, paragraph 2: $\delta_n^{-1}\log(\nu_n)$ should be $\delta_n^{-1}\log(\nu_n^{-1})$.
7. P7, paragraph 3: $V_2 e^{\lambda_1} t$ should be $V_2 t e^{\lambda_1 t}$.
8. P7, paragraph 4: $r_n = n-1$ should be $r_n = n$.
9. P7, paragraph 4: Is it $p0$ or p_0?
10. P10, caption of Figure 4: Probability to detect or of detecting.
11. P13, paragraph 2: The reference to equation (8) on pages 13 and 14 is not correct.
12. P14: In the proof of Proposition 4.1, $-r_n$ and $-r_{n+1}$ should be $-r_n+1$ and $-r_{n+1}+1$.
13. P15, paragraph 4: The period that should be before "The distribution of V_n ..." is on top of the T.
14. P16: In the statement of Proposition 4.5, $-r_n - 1$ should be $-r_n + 1$.
15. P17: In the definition of $\zeta_{n+1}(t,z)$, $Y_{n,1}(t)$ should be $Y_{n+1,1}(t)$ to be consistent with expression (8).
16. P17: The sum symbol in the middle of the page is missing a lower limit.

17. P17: The subscript $n+1$ is missing on multiple letters in the calculations on page 17 (see ζ, λ and ϕ in various places).
18. P20: At the beginning of the proof of Lemma 4.9, γ_n should be δ_n/δ_{n+1} not δ_n/γ_{n+1}, since all cases are being considered.
19. P21: In the final expression in the proof of Lemma 4.9, $\phi^{1-\gamma}$ should not appear in the denominator.
20. P21: After the first "Hence" on page 21, a parenthesis is missing in the denominator. After the second "Hence", "$\mathcal{F}_{n-1}(\boldsymbol{\nu}))^{1/\gamma_n}$" should be "$\mathcal{F}_{n-1}(\boldsymbol{\nu})^{1/\gamma_n})$".
21. P22: In the second display, a parenthesis is missing before the first 1.
22. P22: In the statement of Lemma 4.10, $n > 1$ should be $n \geq 1$.
23. P23: In the first equation, $f_n(\nu_n)$ should be $f_n(\nu_n)^{-1}$.
24. P24: $(u + \sigma_n - \rho_n)^{r_n - 1}$ has an extra parenthesis.
25. P25: In the third display, the last limit should be $\nu_{n-1} \to 0$, not $\nu_n \to 0$.
