# Supplementary material for: Sequential mutations in exponentially growing populations
Source: PLoS Comput Biol. 2023 Jul 10;19(7):e1011289. doi: 10.1371/journal.pcbi.1011289 (PMC10359018; doi:10.1371/journal.pcbi.1011289)
Supplement: Attachment — Submitted filename: PlosCB_referee_response.pdf [file pcbi.1011289.s005.pdf]

April 18, 2023

Dear Professor Foo,

We would like to sincerely thank both reviewers for their careful, thoughtful, and highly constructive feedback on our paper. We believe the reviewers' comments have considerably improved the manuscript. While the paper has been altered significantly, some particular changes we would like to highlight are:

- We have replaced our "Illustrative examples" section with a section fully devoted to mutation rate inference in a fluctuation assay setting. While our results have varied applications outside this specific scenario, we felt that fleshing out one particular example, which has strong experimental motivation, and demonstrating how the results could be used for inference in this example, would best address the reviewers concerns about the applicability of results.
- We have chosen to emphasise that we study sequential mutation accumulation, both by altering the title of the paper and by numerous changes throughout the manuscript. We have added a subsection on the motivation of this setting in the "Model" section.
- We have considerably restructured the paper in order to improve readability, to provide some intuition for the mathematical results, and to extend the comparison with prior work.

We have typeset all changes in red in the manuscript for clarity. A detailed point-by-point response is given below. We would like to thank the reviewers once again for their time and expertise, and look forward to their feedback.

Kind Regards,
Michael Nicholson, David Cheek, and Tibor Antal

1 Reviewer 1

In this manuscript, the authors study a multitype branching process model of mutation accumulation in an exponentially growing cell population. In the model, type- n cells can divide, die or mutate to type- $(n+1)$ at rates α_n, β_n and ν_n, respectively. The authors present mathematical results on (i) the size of the type- n population $Z_n(t)$ at time t, and (ii) the arrival time τ_n of the first type- n cell. For the case where mutations do not lead to fitness increases, $\lambda_i \leq \lambda_1$ for all $i = 1, \ldots, n$, a distribution for $Z_n(t)$ is given for large t and general $\nu_1, \ldots, \nu_{n-1}$, and a distribution for τ_n is given in the limit of small mutation rates (the ordered limit $\lim_{\nu_1 \to} \cdots \lim_{\nu_{n-1} \to 0}$). For the alternative case $\lambda_i > \lambda_1$ for some i, the authors turn to an approximate version of the model. In this

model, the emergence of type- $(i+1)$ cells is studied under the assumption that the type- i population grows according to its long-run trajectory. For example, if $Z_1(t)e^{-\lambda_1 t} \to V_1$ as $t \to \infty$ for type- 1 cells, type- 2 cells are produced at rate $\nu_1 V_1 e^{\lambda_1 t}$ at any time $t \geq 0$. The authors compute the distributions of $Z_n(t)$ for large t and τ_n under the approximate model, in the limit of small mutation rates. An analogous approximate model has been previously applied by Durrett and Moseley to study the case $\lambda_1 < \lambda_2 < \cdots < \lambda_n$.

The main text consists of a summary and discussion of the mathematical results, followed by a brief discussion of their potential application to mutational dynamics in bacteria and cancer. The methods section, which is around twice as long as the main text, contains proofs of the mathematical results. While I think the manuscript is generally well written and the results are interesting, I have the following main concerns:

1. The goal of the paper is to study the distributions of the size of the type- n population and the time of first appearance of a type- n cell in a general setting. The results are discussed as if they hold for any birth and death rates, and Figures 3 and 4 show examples where $\alpha_i < \beta_i$ for some i. However, the proofs seem to assume that each cell type follows a supercritical branching process, with $\alpha_i > \beta_i$ for all i.
   We thank the referee for spotting this. We only need $\alpha_1 > \beta_1$, so we removed other unnecessary statements.
2. For the $\lambda_1 < \lambda_2 < \cdots < \lambda_n$ case, the main results of the paper have already been established by Durrett and Moseley. Insights emphasized in the present manuscript, such as the fact that the shape of the distribution of τ_n is the same for all n, and the fact that the time between new mutants appearing shortens with increasing n for the case $\lambda_1 < \lambda_2 < \cdots < \lambda_n$, have been noted before. The novelty of the results and insights should be addressed more clearly, and the formulas obtained should be compared directly with the formulas obtained by Durrett and Moseley.
   We have added a paragraph discussing the novelty of our results compared to Durrett-Moseley. We have also added some comments on the different notations we use, and that our formulas match in that special case. (minus 2 misprints in the original Durrett-Moseley, which were corrected later in Durrett's summary article.)
3. The authors structure the paper in a way to make it more accessible to nonmathematical readers. However, there is still room for removing technical language, adding context and providing intuition. I make some suggestions below.
   We have tried to add more intuition to the paper, and consider the referee's specific suggestions below.
4. Irrespective of the last comment, I am not sure this manuscript is appropriate for PLOS CB. The mathematical results and their proofs are the meat of the manuscript, whereas the application section lacks develop-

ment. One potential way of addressing this is to flesh out more concretely how the results can be useful for parameter inference or analysis of data, and to preferably show that the ideas work on simulated or real data. A stronger case for the utility of the results seems especially important given that the mathematical contribution and related insights seem to largely extend or reemphasize prior work.
We believe that we provide useful results on a fundamental model that is widely adopted within the computational biology community (in particular, but not exclusively, quantitative researchers focusing on cancer and bacterial evolution). In our revised manuscript we show how our results can be used for inference for fluctuation assays which are used to study both cancer and bacterial mutation rates. We present results for a population of cells undergoing a sequence of mutations where the mutants have arbitrary fitness values (apart from the initial type which needs to be supercritical). We recover two special cases: when fitness increases at each mutation step (Durrett-Moseley), and when the initial cell is the fittest (Nicholson-Antal). These special cases correspond to quite restricted parameter regions relative to our general results. We have provided further discussion on the motivation for studying this model and given a detailed application on fluctuation assays.

5. The manuscript should be read more carefully. I found a significant number of apparent typos, most of them in mathematical expressions.
Many thanks for this.

I make more comments below, split into "major comments" (most of which elaborate on the above comments), "minor comments", and "typos".

1.1 Major comments.

1. Abstract: Referring to the distributions of the number and arrival time of type- n cells, it is said that: "Surprisingly, these distributions are universal in that their shape does not depend on the number or selective effect of the mutations considered." This doesn't seem to be true for the distribution of the number of type- n cells, which requires knowledge of the entire sequence $\lambda_1, \ldots, \lambda_n$.
We have clarified the specific sense of universality, that the number of cells with n mutations and the arrival time of the first cell with n mutations respectively follow Mittag-Leffler and logistic distributions.

2. P4, paragraph 1: I suggest expanding on the discussion of the result in (1). For example, point out explicitly that when $\lambda_n > \delta_{n-1}$, the growth is exponential with rate λ_n, while there is a geometric factor $t^{r_n - 1}$ when $\lambda_n \leq \delta_{n-1}$. In addition, provide intuition for why the geometric factor appears and why the exponent is $r_n - 1$.
We have added a discussion on this.

3. P5, paragraph 1: I suggest expanding on the comment "For $\gamma < 1$ the density has a $x^{\gamma-1}$ singularity at the origin and a $x^{-\gamma-1}$ tail." Discuss the properties of the Mittag-Leffler distribution at a higher level, interpret what these properties mean for the distribution of $Z_n(t)$, and explain why the model dynamics produce a limiting distribution with these properties.
We have added a discussion.

4. P7, paragraph 1: The comment on identifying whether a phenotype of interest requires 2 or 3 mutations based on fluctuations in τ_n presupposes that the distribution of τ_n is observable. Is that the case? Are there other observables that could be used to infer how many steps are required to produce a given phenotype, when combining the theoretical results with data?
We have replaced this example with the more standard fluctuation assay goal of mutation rate inference. While we believe our original comment on identifying whether 2 or 3 mutations are required has merit, we have decided that our paper was not the best place for introducing a new experimental assay for this goal.

5. P7, paragraphs 2-3: I suggest rewriting these paragraphs using more informal language and adding explanations. For example, I am not sure the following sentence is easily parsed by the general reader: "Then, solving for $t^{(2)}_{1/2}$ in $e^{\lambda_1 t^{(2)}_{1/2}} = 1/\nu_1$, produces the leading order as $\nu_1 \to 0$ (to be compared with Eq. (5))".
We have rewritten this part.

6. P7, paragraph 4: In the first application example, an experiment of multidrug resistance is suggested to infer the product $\prod_{i=1}^n \nu_i$ of the rates $\nu_1, \ldots, \nu_n$ for n sequential resistance mutations. I am not sure how realistic this example is. For example, in the hypothetical experiment, what is the time scale of waiting for n sequential mutations? Do the cells need to be repassaged, meaning uninterrupted exponential growth cannot be assumed? Does the appearance of the $\log\left(\nu_n^{-1}\right)$ factor in $t^{n+1}_{1/2}$ interrupt inference of $\prod_{i=1}^n \nu_i$? Even if $\prod_{i=1}^n \nu_i$ is theoretically identifiable by inverting expression (4), how confidently can it be inferred from real data?
As mentioned above, we have replaced this example.

7. P8-P9: In the application examples (ii)-(iv), arrival times of mutations are discussed depending on whether the mutations are advantageous, neutral or deleterious. The qualitative insights are for example that for $\lambda_1 < \cdots < \lambda_n$ and $\nu_1 = \cdots = \nu_n$, the time between the first type- n and the first type-$(n+1)$ cell decreases as n increases, and that the time between the arrival of a new mutant and the mutant growing to a detectable size is greater if the mutation is neutral or deleterious than if it is advantageous. These insights are not necessarily surprising, and in general the examples do not give concrete suggestions of how the theoretical results can be useful for example for statistical inference or analyzing data.
We have discarded applications (iii) - (iv), however have kept a comment

on the increasing rate of evolution when comparing with prior work. We also highlight Bozic et al in our motivation section who do use this model for statistical inference with cancer genetic data. Further, we have added a fluctuation assay section with inference on simulated data.

8. P11: In Lemma 4.2, which is used to prove Proposition 4.1, it is explicitly assumed that $\alpha > \beta$, which is not reflected in the statement of Proposition 4.1.
   We thank the reviewer for spotting this. This assumption of $\alpha > \beta$ is actually not required in this Lemma and has been removed.

9. P17: In stating the limit of the birth-death process $Y(\cdot)$ and its Laplace transform, it is implicitly assumed that $\alpha_{n+1} > \beta_{n+1}$, but this assumption does not appear in the statement of Corollary 4.6 or discussions of this and related results.
   We disagree that we implicitly assume that $\alpha_{n+1} > \beta_{n+1}$, although due to a now-corrected mistake in the statement of Lemma 4.2 (when we stated that λ is required to be positive), we understand why the reviewer believed we make that implicit assumption. Specifically for Corollary 4.6, the limit of the birth-death process is only required for the case $\lambda_{n+1} > \delta_n$. But if $\lambda_{n+1} > \delta_n$ then $\lambda_{n+1} > 0$ as $\delta_n \geq \delta_1 > 0$. Hence in that case we were assuming $\alpha_{n+1} > \beta_{n+1}$, although admittedly obscurely. We have added a comment in the proof of Corollary 4.6 making this clear.

1.2 Minor comments.

1. P3, paragraph 2: It could help to explain what the rates mean, for example in terms of the probability of something happening in a small time interval, since not every reader may be familiar with the continuous-time branching process framework.
   We've added more explanations.

2. P3, paragraph 5: I suggest replacing the semicolon with a period.
   Done.

3. P3, paragraph 5: Immediately before expression (1), I suggest adding something like "and how many times it has been obtained up to type n " after "by the running max fitness".
   Thanks for this suggestion, it has been adopted.

4. P4, paragraph 2: I recommend showing the Laplace transform before introducing the distribution as Mittag-Leffler, since many readers may not know this distribution (including myself). Similarly, on page 5, I would show expression (4) before I call the distribution logistic.
   We have rewritten the presentation of these distributions. We show the Laplace transform for M-L where we introduce it, and show the logistic distribution right where we mention it.

5. P4, paragraph 2: My context-free interpretation of "fluctuations in cell

number" is fluctuations over time, as opposed to variability at a fixed time across many tumors. I wonder if there is a better phrase which lends itself more to the second interpretation (referring for example to the "randomness", "stochasticity" or "variability" in cell number), or if it's worth it to explicitly make this distinction in the text.
We have altered "fluctuations" to "stochasticity" following the referee's suggestion

6. P5, paragraph 3: Is it not relevant to also discuss the time to the first type- n which does not die out?
We have added a comment on this case.

7. P7, paragraph 3: This paragraph is somewhat out of place. I recommend moving it so that it appears closer to the statement of the relevant result (1) and before you start discussing the results for the time to the first type- n. I also suggest splitting the "Results summary" section into two subsections, one on the number of type-n cells at time t, and the other on the arrival time of type- n.
We have restructured the results in line with the reviewers suggestion.

8. P7, paragraph 3: At the end of the paragraph, do you mean $\delta_n - \lambda_{n+1} \ll 1$ as opposed to $\delta_n - \lambda_{n-1} \ll 1$? It's not necessarily accurate to call the n-th mutation a deleterious mutation in this case, since we might have $\lambda_n < \lambda_{n+1} < \delta_n$.
We thank the review for catching this typo, it has been corrected.

9. P10, caption of Figure 4: The end of the caption is confusing, where β_n is set to $1 + ns_d$, whereas earlier in the caption β_1 is fixed to 1.

10. P11: In the statement of Proposition 4.1, the conditioning on nonextinction of type-1s should be explicitly stated.
Done

11. P11: In the statement of Lemma 4.2, and in other similar cases, it would help to state that $Y_i(0) = 1$
We have added this in Lemma 4.2 and the proof of Corollary 4.6.

12. P11: As far as I can tell, there is no argument for the positivity of V in the proof of Lemma 4.2.
We have added a proof of the positivity of V

13. P12: Add "for $s \leq t$ " before the martingale calculation.
Done.

14. P12: When giving the result for the second moment of a birth-death branching process, say where within Harris the result can be found.
We have edited this to a reference in Athreya-Ney as the result was most clear there. We have added the page number and equation.

15. P15: Proposition 4.4 would be more appropriately called a corollary.
This has been altered.

16. P16: If you condition on V_n^*, is the resulting process not simply a Poisson

process? Is there any reason to use the term "Cox process"?
Good point, as we say "given V_n^*" this is indeed a Poisson process. We have altered this accordingly.

17. P17: In the statement of Corollary 4.6, why not simply write $(r_n - 1)\,!$ instead of $\Gamma\,(r_n)$?
We agree, this has been altered.

18. P17: Does the proof of Corollary 4.6 not use the same argument as in the proof of Theorem 3 in Durrett and Moseley? If so, it should be acknowledged.
We have added an acknowledgement.

19. P17: The derivation of the Laplace transform in (10) at the beginning of the proof is a little confusing. $Z_{n+1}(t)$ is a compound Poisson random variable, but it requires a little thought to see that it can be written as a sum of i.i.d. random variables. It would probably help to provide a more explicit calculation showing how one goes from the definition in (8) to the Laplace transform given in (10). A similar comment applies to the proof of Lemma 4.2, where it would also be helpful to show how $Z(t)$ can be written as a sum of i.i.d. random variables.
We have added an additional comment to the proof of Corollary 4.6, and references to the proofs of Lemma 4.2 and Corollary 4.6.

20. P18: For the integral representation of the Lerch transcendent to converge, I think you also need $Re(s) > 0$ and $Re(a) > 0$ to avoid problems at 0 and ∞.
We added this condition for the integral representation. For Lemma 4.8, we use $0 < a < 1$, and we specified that we only consider positive integer values for s.

21. P18: "Recall that it was heuristically argued that the random amplitudes of the approximate and original models coincide in the limit of small mutation rates". On page 16 , it says that "We hypothesize but do not prove that ...", but I don't see a heuristic argument.
Our heuristic argument is in the first paragraph of Section 4.2, directly following the sentence "The approximate model is motivated by the following heuristic argument:"

22. P19: It would help the reader a lot to number the definition of κ_n and refer to it when it is used.
We agree and have followed the reviewers suggestion.

23. P19: In the statements of Proposition 4.7, Lemma 4.10 and what follows, why not write α_1/λ_1 instead of $(1 - \beta_1/\alpha_1)^{-1}$? (This is done in the main text when writing down the recursion for ω_n.)
We agree and have followed the reviewers suggestion.

24. P19: Is there no standard reference for Lemma 4.8?
It is discussed in Theorem 3 of Chelo Ferreira, Jose L. Lopez: Asymptotic expansions of the Hurwitz–Lerch zeta function, J. Math. Anal. Appl. 298

(2004) 210–224, but it unfortunately contains an incorrect step and hence gives a wrong result. That method couldn't be corrected, hence we came up with our own expansion with help from colleagues with expertise in special functions (Prof. Olde Daalhuis) on this matter.

25. P20: In the statement of Lemma 4.9, before showing the three cases, it would help the reader a lot to write the result more compactly as

$$h_n \left(f_n \left(\nu_n \right)^{1/\gamma_n} \theta \right) = \theta^{\gamma_n} \kappa_n.$$

Having this form prominently displayed in the statement of the lemma helps when reading the proof of Proposition 4.7 on page 21 .
This has been added

26. P21: What are called terms at the top of the page are factors.
This has been altered

27. P22: I would suggest using a letter other than V_{n+1}, since V_{n+1} is used in a different context in the statement of the earlier Proposition 4.1.
We thank the reviewer for this suggestion, the standard notation of V_{n+1}^* is now used here.

28. P22: In the calculations for ω_{n+1} in the proof of Lemma 4.10, it would be more logical to reverse the two steps taken. The same comment applies to the calculations for ω_{n+1} on the following page.
We thank the reviewer for pointing out that the logical flow of this proof could be improved. We have rewritten the proof.

1.3 Typos.

1. General: There are multiple places where punctuation marks are off (a period is missing, a comma appears where a period should be, etc.).
We have corrected many instances of missing punctuation.

2. General: There are frequently double spaces following a period which does not end a sentence. For example, see the space between i.e. and δ_n on page 3, paragraph 3 . This can be avoided by using tilde in the Latex code: "i. e. $\$\backslash delta = n\$$".
Corrected where we've found the issue (although for the particular cited example of "i.e." and δ we're not sure the tilde made much difference)

3. P3, paragraph 3: In the definition of r_n, there is a typo in $i = 1, \ldots, n$.
Corrected

4. P4, caption of Figure 1: "with dashed line" should probably be "with dashed lines".
Corrected

5. P5: In the top line, $n > 1$ should be $n \geq 1$.
Corrected

6. P7, paragraph 2: $\delta_n^{-1} \log \left(\nu_n \right)$ should be $\delta_n^{-1} \log \left(\nu_n^{-1} \right)$.

Corrected

7. P7, paragraph 3: $V_2 e^{\lambda_1} t$ should be $V_2 t e^{\lambda_1 t}$.
Corrected
8. P7, paragraph 4: $r_n = n - 1$ should be $r_n = n$.
Agree, but this sentence has been rewritten
9. P7, paragraph 4: Is it $p0$ or p_0 ?
We now consistently use p_0.
10. P10, caption of Figure 4: Probability to detect or of detecting.
Corrected
11. P13, paragraph 2: The reference to equation (8) on pages 13 and 14 is not correct.
Corrected
12. P14: In the proof of Proposition 4.1, $-r_n$ and $-r_{n+1}$ should be $-r_n + 1$ and $-r_{n+1} + 1$.
Corrected
13. P15, paragraph 4: The period that should be before "The distribution of $V_n \ldots$. is on top of the T.
Corrected
14. P16: In the statement of Proposition 4.5, $-r_n - 1$ should be $-r_n + 1$.
Corrected
15. P17: In the definition of $\zeta_{n+1}(t,z), Y_{n,1}(t)$ should be $Y_{n+1,1}(t)$ to be consistent with expression (8).
Corrected
16. P17: The sum symbol in the middle of the page is missing a lower limit.
Corrected
17. P17: The subscript $n+1$ is missing on multiple letters in the calculations on page 17 (see ζ, λ and ϕ in various places).
Corrected
18. P20: At the beginning of the proof of Lemma 4.9, γ_n should be δ_n/δ_{n+1} not δ_n/γ_{n+1}, since all cases are being considered.
Corrected
19. P21: In the final expression in the proof of Lemma 4.9, $\phi^{1-\gamma}$ should not appear in the denominator.
Corrected
20. P22: In the second display, a parenthesis is missing before the first 1.
Corrected
21. P22: In the statement of Lemma 4.10, $n > 1$ should be $n \geq 1$.
Corrected
22. P23: In the first equation, $f_n(\nu_n)$ should be $f_n(\nu_n)^{-1}$.
This has been rewritten.

23. P24: $(u + \sigma_n - \rho_n))^{r_n - 1}$ has an extra parenthesis.
Corrected

24. P25: In the third display, the last limit should be $\nu_{n-1} \to 0$, not $\nu_n \to 0$.
Unfortunately we don't know that the reviewer is referring to "In the third display". We corrected a ν_n to be a ν_{n-1} in the proof of Corollary 4.5, which is perhaps what the reviewer refers to.

2 Reviewer 2

Nicholson et al. studied the probability distributions of the arrival time and abundance of mutants in an exponential growing population. They reported the shapes of these distributions are independent from the selection strength and the number of the mutations under small mutation rates.

My main question is about the linear evolutionary path assumption. The definition of net growth rate seems to be specific for type n cells with n particular mutations. This differs from standard random mutation accumulation processes, where cells with n mutations can have different combinations of mutation set. The linear evolutionary path seems to be a strong assumption related to a very specific regime of driver mutations, where a new mutation only arises in cells containing all previous mutations. Consequently, the rate of new mutation arising in the whole population is not related to the total population size or total cell numbers under reproduction, but only to the sub population size of the last mutant. It might appears in biological data that driver mutations are detected one after another over time, which is an outcome under the balance of selection and fixation. But I think this is different from the assumption of the sequential acquisition of driver mutations in this model. It would be clear if the authors can cite some experimental papers beside of pervious theoretical models to specify under which context this assumption is relevant. It makes it hard to understand the generality of the results without understanding the motivation of this assumption.

Alternatively If possible, it would be good to know under which condition a standard natural mutation accumulation process would lead to the linear evolutionary path patterns as in this paper.

We have added a discussion of the motivation and relevance of our model in the Model section. We have also added an fluctuation assay inference section illustrating one application of the results. Further, to clarify the generality of the results we have slightly altered the title to reflect our main focus on sequential mutations.

Some results are obtained under certain special conditions compared to the general set up of this model. Can you group those results with more clear subtitles? For example, I was not sure whether eq 3) still belongs to the condition that

---

"type 1 has the maximal growth rate of all types up to type n, that is $\delta_n = \lambda_1$ ".

We have grouped together our results into Population sizes and Arrival times, and have tried to be more clear when a result holds under a condition.

Relatedly, it is better to add some intuitive explanation of some conditions, e.g. "If we assume each running-max fitness is attained only by one type ($r_i = 1$ for each i) ". If we think about a curve representing the net growth rates of all types up to type n with the x-axis representing type $1, 2, \ldots n$, what kind of shapes such a curve should be to fulfil this condition? What does it mean biologically?

We have added a supplementary figure illustrating the curve that the referee suggests. We use this curve to illustrate biologically relevant examples, and also to highlight the novelty of our work compared to prior work.

Fig 2 shows some stochastic simulations under certain λ_1/δ_2 values constructed by given α_i, β_i, ν_i. Does the concrete choice of α_i, β_i, ν_i have any impact on outcomes of the stochastic simulations?

Across a range of parameters we've found our analytic results to match well with exact simulations. Fig. 2 shows this over a range of parameters for the number of cells of type n. We have added further simulations showing the analytic results for the arrival times hold over a broad parameter range also (see Fig. 6).

Minor Comments:

1. There are many notations. I was rolling up and down to follow them. It might be helpful to have a table of all notations.

We have added a table.

2.Page 8 Figure 1 caption: "Grey line indicates where the population sizes". I think you did not mean the total population size here. This can be more specified.

We have rewritten this to improve clarity.

3.. The definition of V_n does not follow Eq. 1), where it is first time introduced.

This has been altered following the reviewers suggestion.

4." Similarly to the population sizes, the exact distribution of the arrival time is analytically intractable outside of the simplest settings." what are the simplest settings?

We have added a reference to work that provides an exact solution to the probability type 3 cells have arrived by t.

5. Can you add the details of the stochastic simulations in the method part?

The simulation approach directly follows the newly added sentence under the model description “In other words after a random, exponentially distributed waiting time with parameter $\alpha_n + \beta_n + \nu_n$, a type (n) cell is replaced by one of the listed three options with probability proportional to the corresponding rate”. We have also added a link to a repository where code for the simulations may be found.
