# Supplementary material for: Sequential mutations in exponentially growing populations
Source: PLoS Comput Biol. 2023 Jul 10;19(7):e1011289. doi: 10.1371/journal.pcbi.1011289 (PMC10359018; doi:10.1371/journal.pcbi.1011289)
Supplement: Attachment — Submitted filename: PlosCB_referee_response_round2.pdf [file pcbi.1011289.s007.pdf]

June 19, 2023

Dear Professor Foo,

We are grateful that our edits have been positively received and would like to yet again thank both the reviewers and yourself for the time and attention given to this paper. In particular, we'd like to thank Reviewer 2 for the extra comments given during this round of reviews. Addressing these comments has improved the paper in our view and crucial typos have been corrected.

In addition to the changes suggested by Reviewer 2, we have also moved supplementary figure S1 to the main text as Figure 2. This was motivated by feedback from colleagues who did not immediately see the novelty of the paper compared with prior work.

We have typeset all changes in red in the manuscript for clarity. A detailed point-by-point response is given below.

Kind Regards,
Michael Nicholson, David Cheek, and Tibor Antal

1 Reviewer 2

I thank the authors for taking my comments seriously and for making a substantial effort to address them. The structure of the manuscript has improved and intuitive explanations and clarifications have been added which have made me better understand and appreciate the results. For example, I believe it is important to say and explain that only the first cell type needs to be supercritical for the arguments to work, as is done clearly now. There are also nice explanations of how the $t^{r_n - 1}$ factor arises and the heavy tailed nature of V_n. More detailed comparisons to previous work have been added. I finally appreciate the new application section which is much more concrete than the previous one. In summary, both my major and minor concerns have been addressed well.

I do have several minor comments on the revision, which I believe the authors will be able to address quickly. The comments are split into "Minor comments", "Writing suggestions" and "Language and typos". Many of the comments are offered as suggestions to further improve the readability of the paper.

Overall, I think this is a nice manuscript which will be suitable for publication in PLOS CB once the comments have been addressed.

Minor comments:

1. P3, paragraph 4: In the examples given in the "Motivation" section, it's

generally unclear when the type-1 should be seen as wild-type (mutation-free), and when it should be seen as the first cell that has undergone an alteration. In the colorectal cancer example, a cell with the first driver is considered type-1. In that case, the waiting time to a cell with 3 driver mutations is relative to the appearance of the cell with the first driver. This also assumes that there is no parallel evolution, where cells with the first driver mutation continue to emerge from the wild-type tissue as the primary tumor clone is evolving. I know the type-1 designation is application-dependent, but I wonder whether it is worth it to point out that it is possible to treat type-1 as either wild-type or an altered cell, and to discuss the implications of each choice. It's useful for the reader I think to have in mind that both choices may be warranted depending on the application.
We have added "The cancer evolution examples discussed above all assume that the type 1 cell has a driver mutation. In other settings, it may be more natural to consider the type 1 cells as wild type, for example when considering the emergence of drug resistance. We emphasise that in this paper the type one cells are always supercritical, that is they grow exponentially on average.". The reviewers point about parallel evolution from healthy tissues is important, and in fact our results can be used as an important stepping stone to treat that more general case, for example by using the arrival time probabilities to thin an initiation process from healthy cells. However, we would prefer not to go into detail on this here as it would distract from the narrative.

2. P4, paragraph 1: I find the sentence starting "Whether to ..." confusing. Can you rephrase it to make it clearer what is done in [14]?
We have rewritten these sentences, but would prefer not to go into more detail on the work of [14] as the biology and sequencing considerations are difficult to summarise succinctly.

3. P4, paragraph 2: When you say you recently derived simple formulas for the arrival time and cell number through any "subset of paths" in the directed graph of genotypes, what does that mean exactly? Do the formulas hold for any path in the directed graph, or do you derive formulas that are dependent on the specific subset of paths being investigated? Are specific subsets of more interest than others?
We would prefer not to go into detail on these points in the paper, and so as to avoid confusion we have changed this sentence to "When the original cell type has the largest net growth rate, we recently derived simple formulas for the arrival time and cell number through the directed graph of genotypes ". But to answer the reviewers questions: the set up of Ref 11 was a graph with a root vertex, where the population was initiated, analogous to type 1, and a target vertex, representing a specific genotype of interest, e.g. a cell with n mutations conferring resistance to n drugs. Then, for a node on this graph, corresponding to a population state of cells with k out of n mutations, the distribution of the population size

and arrival times could be obtained, as well as the probability of arriving at that vertex via any subsets of paths (a subset of all the paths from the root vertex to the selected vertex through the graph). Interestingly, the shape of the distributions did not depend on the subset of paths selected, but the parameters of those distributions did, in particular depending on the mutation rates between types and growth rates of types along the paths. Certain paths may be of more interest, if, e.g., therapy resistance is believed to be acquired via a mutation to gene A, followed by B, followed by C.

4. P5, paragraph 2: I wonder whether it might be good to reference the three subsections of Section 3 at the start of Section 3. In particular, this lets the reader know that there is a separate subsection (Section 3.2) where the main results are interpreted, which is good to know when one reads Section 3.1.
   We have added a sentence indicating there are 3 sections to come in the results.

5. P8, paragraph 2: I recommend saying that " c_n is as in (2)" after expression (7).
   Altered

6. P10, paragraph 3: In the sentence starting "Then we simply find ...", say first that here you are considering the case $n = 1$.
   Altered

7. P11, paragraph 1: The sentence starting "For a comparison ..." is a little confusing. Can you rephrase? (Also, note the "the the" in this sentence.)
   Altered

8. P11, paragraph 2: Can you rephrase the following sentence to clarify its meaning: "However, some modelling assumption may be invalid if multiple amplifications are required for resistance". Are you just saying that it's not valid to assume a single mutational transition to resistance in this case?
   Amended to "However, the modelling assumption of a single mutation imbuing therapy tolerance may be invalid if multiple amplifications are required for resistance."

9. P12, paragraph 1: For the inference, one appears to need to know the birth and death rates of type 1. Can you say that explicitly and comment on how these would be obtained?
   We have added "Note that our inference here has assumed known birth rates and no death. These rates could be measured by standard experimental protocols, for example using growth curve assays." While there are more specific methods to measure birth and death rates in cell culture, these can depend on the cell type considered (bacteria/yeast/mammalian) and as we are not experts on this topic we would prefer to not go into more detail on this.

10. P16, paragraph 1: Unfortunately, the referenced formula for the second moment on page 109 of Athreya and Ney is incorrect. I think they are showing $E[Z(t)(Z(t)-1)]$, in which case their formula is consistent with yours. Can you make a comment on this or point to a reference where the formula is correct?
Indeed, we agree with the reviewer that the formula in Athreya and Ney is incorrect. We have now referenced Harris instead.
11. P16, paragraph 1: The formula for the second moment holds for $\lambda \neq 0$. Can you comment on how the proof works out for $\lambda = 0$?
We have added a comment at the end of the proof indicating changes required for the $\lambda = 0$ case.
12. P23, statement of Proposition 5.7: I think that in all instances where you intended to replace $(1-\beta_1/\alpha_1)^{-1}$ by α_1/λ_1, you mistakenly wrote $(\alpha_1/\lambda_1)^{-1}$. Please check this in the statement of Proposition 5.7 and the many following substitutions.
Sincere thanks for catching this error which would have been very difficult to parse for future readers. This has been corrected.
13. P27, paragraph 1: I find the logic of the proof of Lemma 5.10 confusing. It's written as if (19) is known to be true and (18) is to be proven, whereas the opposite is true. For example, for the case $\delta_n \geq \lambda_{n+1}$, instead of assuming that $\omega_{n+1} = \nu_n \kappa_n \omega_n$, does it not make more sense to show that $\nu_n \kappa_n \omega_n = \omega_{n+1}$? I think this is essentially what's being done in the proof, even though it's written in a strange way.
We agree and have restructured the proof as suggested.
14. P33, paragraph 1: The sentence starting "Note that for the simulation parameters ..." is confusing, can you rephrase?
Altered.
15. P33, paragraph 1: It's probably good to say that the procedure you describe gives a 95% confidence interval, which I think is the case here.
Altered.

Writing suggestions:

1. P3, paragraph 2: I suggest to start a new sentence when you introduce δ_n.
Altered.
2. P3, paragraph 4: I suggest to remove the sentence starting "Knowing how ...". This point has been made before and it distracts from the overall point of the paragraph, which is to give an example of a linear evolutionary path.
Altered.
3. P3, paragraph 5: I suggest to move the sentence starting with "Conversely to" into a new paragraph.
We'd prefer not to split this paragraph as the resulting paragraphs would

be small and it currently has a coherent message, that is modelling of positive and negative selection in cancer.

4. P4, paragraph 2: Reword the sentence starting "In the results presented below" to something like: "The results presented below, where the cell type with the largest net growth rate is unconstrained, hold only for a linear path through a genotype space."
   Altered.

5. P6, paragraph 1: The first paragraph is a little bit dizzying since there is so much information presented in sequence. Some suggestions for improvement:

- Remove the sentence starting "Notably, when type 1 ..." before "Stochastic simulations ...", and merge this sentence with the later sentence starting "For $\gamma = 1$ its density is simply $e^{-x} \ldots$" since it makes a similar point.
  We absolutely see the reviewers point. However we would like to keep the first sentence as the latter sentence states that V_n/ω_n is a unit one exponential when $\gamma = 1$, whereas the former is explicitly about the random amplitude V_n. While each sentence implies the other, we'd prefer to not suppress the properties of the more important quantity which is the unscaled random amplitude.

- Start a new paragraph at the sentence starting "The variable $V_n/\omega_n \ldots$.
  Altered, and we agree it very much makes an improvement for readability

6. P7, paragraph 1: Instead of the parentheses, I suggest starting a new sentence with "For example, the exact probability that type 3 cells ...".
   Altered.

7. P9, paragraph 2: The intuition provided in the first two paragraphs of Section 3.2 is nice, thank you for adding those. However, the second paragraph in particular could be written a little bit better. Some suggestions:

- Replace "potentially with infinite mean (if $\lambda_1 < \delta_n$) driven by a power-law decay in its distribution" with "with infinite mean if $\lambda_1 < \delta_n$ driven by a power-law decay in the tail of the distribution." In general, I think it's better to avoid parentheses if you can.
  Altered.

- Add "In this case, the tail is exponential." before the sentence starting "To move to type $n \ldots$.
  Altered.

- I'm a little confused by the last three sentences. You say that for $\delta_n \geq \lambda_{n+1}$, the randomness in cell number is inherited between type n and $n+1$, and then that if there is an i such that $\delta_i < \delta_{i+1}$, a power-law tail emerges and is propagated for $j > i+1$. Is this written with the understanding that $\delta_j = \delta_{i+1}$ for $j > i+1$? I recommend rewriting these sentences for clarity.

We have significantly rewritten this section to provide further clarity.

8. P12, paragraph 2: Start a new sentence at "Instead, the long-time limit ...". Altered.

Language and typos:

1. In the abstract, I think "consequences on" should be "consequences for". have altered this to "We highlight consequences for mutation rate inference in fluctuation assays."
2. P2, paragraph 3: "the effects of ... remain unclear" (remains $\rightarrow$ remain). Corrected.
3. P3, paragraph 1 and P7, paragraph 1: Replace "type (n) " and "type $(n+1)$ " by "type n " and "type $n+1$" ", to not be confused with the special notation (n). This is also generally the convention in the text. Corrected.
4. P3, paragraph 2: I think it makes more sense to say that the running max fitness has been "attained" than "obtained". 5. P3, paragraph Corrected.
5. "such that each new mutations" $\rightarrow$ "such that each new mutation". Corrected.
6. P4, figure caption: Remove the first "line" from "Grey line horizontal line". Corrected.
7. P4, figure caption: "grey vertical lines indicates" $\rightarrow$ "indicate". Corrected.
8. P4, figure caption: λ_2 is 0.1 , not 0.2 , in this example. Corrected.
9. P6, paragraph 1: In response to a comment in my original review, I think you erroneously changed $n>1$ to $n \geq 1$ before expression (7), whereas you should have done it before expression (2). So now, I think you need to change $n>1$ to $n \geq 1$ before expression (2), and $n \geq 1$ back to $n>1$ before expression (7). Corrected.
10. P7, paragraph 1: In the paranthesis, add "time" before " t ": "type 3 cells arrive by time t". Corrected.
11. P7, paragraph 1: The period at the end of expression (4) should be a comma or have no punctuation mark. Corrected.
12. P8, paragraph 2: I realize that $\delta_{n-1}=\delta_n$ in the first case, but I recommend either consistently writing $\delta_n-\lambda_n$ or $\delta_{n-1}-\lambda_n$ inside the log for this case

in expressions (7) and (24).
Corrected.

13. P12, paragraph 1: Remove extra "the" from the top line.
Corrected

14. P12, paragraph 1: "takes this simple form of" → "take the simple form of".
Corrected

15. P12, paragraph 2: In the final sentence of Section 3, "allows" → "allow".
Altered to 'allow for' which seemed most appropriate.

16. P24, statement of Lemma 5.9: The new statement of the result of Lemma 5.9 should have $\kappa_n \theta^{\gamma_n}$ on the right-hand side, as opposed to just θ^{γ_n}.
Corrected.

17. P24, statement of Lemma 5.9: Remove the period after θ^{γ_n} in the first expression.
Corrected.

18. P24, statement of Lemma 5.9: Add a period at the conclusion of the final expression.
Corrected.

19. P24, paragraph 3: Remove the sentence starting "Note the right hand side of ...", since the new statement of Lemma 5.9 has incorporated this comment. (As an aside, there is also a typo in this sentence, where θ should be θ^{γ_n}.)
Corrected.

20. P27, paragraph 2: Put a period after expression (21).
Corrected.

21. P32, paragraph 5: Write P0 as p_0.
Corrected.

22. P32, paragraph 5: "were we used" → "where we used".
Corrected.

23. P33, paragraph 1: Remove extra "the" from "such that the the".
Removed.
